# Influence of *RAR*α gene on *MDR1 *expression and P-glycoprotein function in human leukemic cells

**DOI:** 10.1186/1475-2867-5-15

**Published:** 2005-05-24

**Authors:** Tatjana P Stromskaya, Ekaterina Y Rybalkina, Tatjana N Zabotina, Alexander A Shishkin, Alla A Stavrovskaya

**Affiliations:** 1 Institute of Carcinogenesis, N.N. Blokhin Russian Cancer Research Centre of the Russian Academy of Medical Sciences, Kashirskoye sh 24, Moscow 115478, Russia

## Abstract

**Background:**

Multidrug resistance (MDR) phenotype of malignant cells is the major problem in the chemotherapy of neoplasia. The treatment of leukemia with retinoids is aimed on the induction of leukemic cells differentiation. However the interconnections between retinoid regulated differentiation of leukemic cells and regulation of MDR remains unclear.

**Methods:**

Four lines of cultured leukemic cells of diverse types of differentiation were infected with *RAR*α gene and stable transfectants were isolated. We investigated the differentiation of these cells as well as the expression of *RAR*α and *MDR1 *genes and P-glycoprotein (Pgp, MDR protein) functional activity in these cells.

**Results:**

All *RAR*α transfected sublines demonstrated the increase in the quantity of *RAR*α mRNA. All these sublines became more differentiated. Intrinsic activity of *MDR1 *gene (but not Pgp functional activity) was increased in one of the transfectants. *All-trans*-retinoic acid (ATRA) induced Pgp activity in two of three infectants to a larger extent than in parental cells.

**Conclusion:**

The data show that RARα regulates *MDR1/ *Pgp activity in human leukemic cells, in the first place, Pgp activity induced by ATRA. These results show that RARα overexpression in leukemic cells could result in MDR.

## Background

Multidrug resistance (MDR) phenotype of malignant cells is the major problem in the chemotherapy of neoplasia. P-glycoprotein (Pgp) activity is recognised to be one of the major mechanisms responsible for MDR. Pgp transports many structurally diverse compounds across the cell membrane and confers the MDR phenotype in tumor cells [1]. A number of signaling pathways participate in the regulation of *MDR1 *gene expression and the activity of its product, Pgp [2]. Some of these signaling pathways could participate in coordinated regulation of *MDR1/*Pgp activity, cell proliferation and cell differentiation. It was shown that retinoic acid (RA) can modulate *MDR1 *gene expression [3-5]. Retinoids are known to be involved into the regulation of the cell growth, differentiation and apoptosis. In the last decade retinoids became implicated into the treatment of leukemia and some solid tumors [6]. This approach changed the focus of the haematological diseases treatment from the cytotoxicity of the anti-cancer drugs to the reversal of arrested maturation of leukemic cells. Retinoids act via two families of receptors (RARs – RARα, RARβ, RARγ) and RXRs (RXRα, RXRβ, RXRγ). There is the evidence that RARα is the crucial receptor mediating the biological effects during retinoid signaling in some cells [7]. Cell differentiation caused by the stable overexpression of receptor RARα was shown to result in constitutive over expression of *MDR1 *gene in some cultured cells of solid tumors [4]. However the interconnections between RA/RARα regulated differentiation of leukemic cells and regulation of *MDR1*/Pgp activity remains unclear. In some leukemic cells RA did not influence *MDR1 *and/or Pgp activity, while in the others it either augmented or reduced *MDR1*/Pgp expression [5,8]. The aim of this study is to investigate if effects of *all-trans*-retinoic acid (ATRA) on *MDR1*/Pgp activity in leukemic cells are connected with *RAR*α expression and with the leukemic cell differentiation. We isolated sublines of cultured leukemic cells characterized by the stable *RAR*α overexpression and investigated the constitutive and ATRA induced *MDR1*/Pgp activity in these cells. Our data show that various *RAR*α transformed leukemic cell lines acquired more differentiated phenotype. Constitutive level of *MDR1 *gene expression increased in one of *RAR*α overexpressing cell sublines. *RAR*α overexpression did not influence Pgp functional activity while Pgp activity induced by ATRA was elevated in all infectants studied. This shows that the main effect of *RAR*α in the cells studied is its influence on the induced functional activity of Pgp.

## Methods

### Cell lines and culture

Lines of cultured leukemic cells used in the study: H9 cells (acute human T-cell leukemia) [9], KG-1 cell line (cells of acute myelogenous leukemia) [10], K562 cell line (cells obtained from the patient in blast crisis of chronic myeloid leukemia) [11], NB4 (acute promyelocytic leukemia) [12].

Cells were grown in RPMI-1640 medium supplemented with 10% fetal calf serum (Gibco, USA), 2 mM L-glutamine, 50 μg/ml gentamycin at 37°C in a fully humidified atmosphere of 95% air and 5% CO_2_. All the derived cell lines described in this paper were obtained by retroviral infection and selection with the appropriate antibiotic. ATRA (*all-trans*-retinoic acid, Sigma, USA) was added to the culture medium at seeding or 24 hours after seeding (see Legends to Figures).

### Expression vector and retrovilal infection

The PA317/LRARSN retroviral vector-producing cell line was used. All the procedure was described earlier [4]. In brief, the vector used contains a cDNA fragment harbouring the complete coding sequence of the *RAR*α gene driven by the Moloney murine leukemia virus long-terminal repeat as well as the SV40 early promoter-driving neomycin phosphotransferase gene (*neo*) as a selectable marker [13]. The cells (4 × 10^5 ^per 25-cm^2 ^flask) were seeded 24 h before infection. Conditioned medium from a retrovirus-producing cell line was filtered through a 0.45-μm membrane (Millipore, USA), diluted 1:1 with medium, containing 1% serum and 8 μg /ml Polybrene and added to the cells for 24 h at 37°C, 5% CO_2_. Further selection were carried out by culturing cells in medium supplemented with 400 μg/ml G418 (Gibco, USA) for at least 21 days. The medium was changed twice a week. The pool of G418-resistant cells was resuspended in culture medium and progressively expanded.

### Assay of cell growth, apoptosis and differentiation

Cells were seeded into 24-well plates (1 × 10^4 ^cell per well) and the cell number was counted at days 1, 3, 5 and 8 after seeding. The apoptosis in the populations of the parental and *RARα *infected cell lines was performed using the standard procedure [14]. Cells were collected 24 h after seeding, washed with PBS, and fixed in 70% ethanol overnight at 4°C. Fixed cells were suspended in citric buffer and stained with propidium iodide (5 mcg/ml) in PBS for 1 hour at 4°C. DNA content was subsequently measured by FACScan (Becton Dickinson, USA).

The immunophenotype of the cells was evaluated as previously described [15]. Surface expression of the following antigens was determined: CD3, CD5, CD7, CD8, CD11b, CD13, CD15, CD33, CD34, HAE3 and HAE9. In brief, cells were incubated with phycoerythrin-labelled monoclonal mouse antibodies for 20 min at 4°C (Becton, Dickinson), washed with RPMI 1640 medium and analyzed with a flow cytometer (Becton Dickinson).

### Analysis of rhodamine 123 (Rh123) efflux by the cells

The technique used in the study was described in [16]. Cells were loaded with 5 μg/ml Rh123 (Sigma) for 10 min at 37°C, washed twice with cold PBS, pH 7.2, and incubated for 30 min in dye-free medium at 37°C. After the completion of incubation, cell were washed twice with cold PBS. Cell fluorescence was measured on a flow cytometer FACScan (Becton Dickinson, USA). Each measurement counted 5000 events. Non-viable cells were gated out of the analysis on the basis of side scatter.

### RNA isolation and reverse transcriptase polymerase chain reaction (RT-PCR) analysis of RARα and MDR1 genes expression

The cells were dissolved in TRI reagent (Sigma, USA). Total RNA was isolated as described in the manufacturer's manual. For analysis, aliquots of isolated RNA were denatured with formamide and subjected to electrophoresis in 1.8% agarose gels. The samples with clearly visualized 18S and 28S RNA bands were used for further procedures. First-strand cDNA was synthesized using reverse transcriptase M-MuLV (MBI Fermentas, Russia) with 4 μg RNA as a template, 2.5 ng random hexamers, 0.25 mM of each deoxynucleotide triphosphate (SibEnzyme, Russia), dithiothreitol, 4 Units of RNAase inhibitor (MBI Fermentas, Russia) and 100 Units of M-MuLV RT. The reaction was performed at 42°C for 50 min, and 1/60 volume of reaction mixture was used for amplification. PCR was done in a total volume of 25 μl using the thermocycler "Tercyc" (DNA-technology, Russia). The PCR mixture consisted of (NH_4_)_2_SO_4_-containing PCR buffer ("MBI Fermentas"), 0.160 mM dNTPs mix ("MBI Fermentas"), 2 mM MgCl2, 20 pmoles of each specific primer and 0.8 Unit of Taq-polymerase ("MBI Fermentas"). PCR was done as follows: 94°C for 2 seconds, Tm (different for each gene) for 10 seconds, 72°C for 5 seconds. Semi-quantitative PCR analysis of *RARa *and *MDR1 *genes expression were performed using oligomers amplifying a 333 bp and 167 bp products, respectively. Specific gene primers used for RT-PCR are given in Table 1. The amounts of template cDNAs were normalized by PCR amplification of β2-microglobulin cDNA (internal control). The optimal numbers of PCR cycles were 24 for the b2-microglobulin, 26 for RARα-specific product, 33 for *MDR1 *(for all cells lines except KG1 and KG1/RAR, for these cells the numbers of PCR cycles *MDR1*-specific product were 26). These numbers of cycles yielded clearly detectable PCR products within an exponential range. PCR products were amplified in separate tubes, resolved by electrophoresis in 2% agarose gel, stained with ethidium bromide and visualized in UV light.

**Table 1:** Specific gene primers used for RT-PCR

Gene	Product size	Primer	Sequence
*RARα*	333 bp	F	5'-GTCTTTGCCTTCGCCAACCAG-3'
		R	5'-GCCCTCTGAGTTCTCCAACA-3'
*MDR1*	127 bp	F	5'-CCCATCATTGCAATAGCAGG-3'
		R	5'-GTTCAAACTTCTGCTCCTGA-3'
*β2m*	114 bp	F	5'-ACCCCCACTGAAAAAGATGA-3'
		R	5'-ATCTTCAAACCTCCATGATG-3'

## Results

### Influence of RARα gene overexpression on cell differentiation, proliferation and spontaneous apoptosis

**Figure 1:**
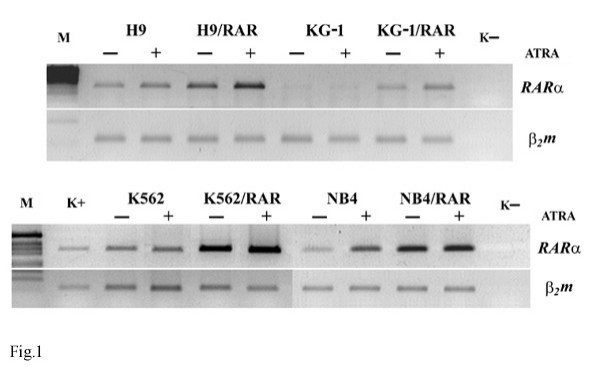
**Expression of *RARα *mRNA in parental and *RARα *transfected cell lines. RT PCRM**. k- – water. ATRA (5 μM) was added to cell cultures 24 h after seeding for 48 h. Then the cells were collected and processed as specified in "Methods" (RNA isolation and reverse transcriptase polymerase chain reaction (RT-PCR) analysis of *RARα *gene expression). This figure is representative of 2 separate experiments.

*RARα *gene was introduced into the cultured leukemic cells of diverse types of differentiation as described in Methods. The sublines of H9, KG-1, K562 and NB4 cells characterized by the capability to grow in the medium supplemented with G418 were isolated (H9/RAR, KG-1/RAR, K562/RAR and NB4/RAR). Semi-quantitative RT-PCR revealed more pronounced expression of *RARα *mRNA in all transfected cell lines in comparison with the wild type cells (Fig. 1). ATRA (5 μM applied for 48 h) increased *RARα *mRNA in some *RARα *transformed cells (H9/RAR, KG-1/RAR, K562/RAR) to a greater extent than in parental cells (Fig. 1).

**Figure 2:**
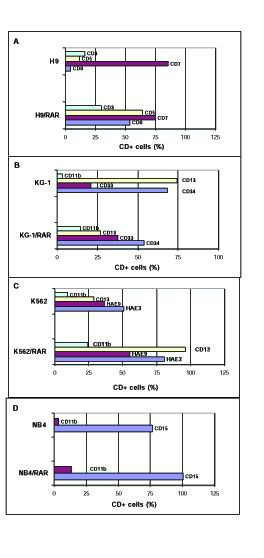
**Comparison of antigen expression by the parental and *RARα *infected cell lines**. Cells were incubated for 30 min at 4°C in the presence of an appropriate monoclonal antibody. After three washes with PBS, cells were incubated for 30 min at 4°C with goat antimouse IgG labeled with phycoerythrin and then analyzed in flow cytometer (Becton Dickinson).

The investigation of the differentiation status of these cells shows that all *RARα *transfected sublines differ from the parental cell populations (Fig. 2). *RARα *transfected H9 culture contains more cell variants expressing CD5 and CD8 antigens than parental cell line (Fig. 2A). Thus the number of cells with antigens of later lymphoid differentiation markers increased in *RARα *overexpressing H9 cells. There is phenotypic evidence of granulocytic differentiation in KG-1/RAR cell subline as indicated by a reduction in CD13 expression and the increase in the expression of CD11b antigen in comparison with parental cells (Fig. 2B). In KG-1/RAR cell population the portion of CD34 cells decreased and the portion of CD33 cells increased (Fig. 2B). This also testifies to increased differentiation of these *RARα *overexpressing cells. In K562/RAR population the number of the cells of erythroid differentiation (expressing HAE9 and HAE3 antigens) is larger than in K562 population (Fig. 2C). Hemoglobin synthesis is increased in K562/RAR culture more than 5-fold in comparison with parental cells (not shown). In NB4/RAR population the number of the cells of myelogenous differentiation (expressing CD11b and CD15 antigens) is larger than in NB4 population (Fig. 2D).

**Figure 3:**
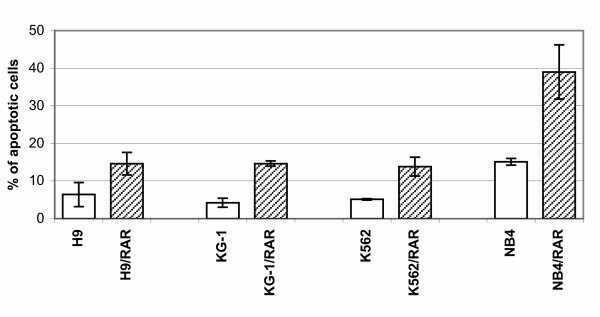
**Spontaneous apoptosis in the populations of the parental and *RARα *infected cell lines**. Propidium iodide flow cytometry detection of dead cells was performed using the standard procedure. Cells were collected 24 h after seeding, washed with PBS, and fixed in 70% ethanol overnight at 4°C. Fixed cells were suspended in citric buffer and stained with 5 mcg/ml propidium iodide in PBS for 1 hour at 4°C. DNA content was subsequently measured by FACScan (Becton Dickinson, USA). All cells with sub-G_0 _DNA content were regarded as dead cells. This figure is representative of 3 separate experiments.

The percentage of cells undergoing spontaneous apoptosis increased 2-3-fold in all *RARα *transfected cell populations (Fig. 3). This could be connected with more differentiated phenotype of *RARα *transformed cells. It seems that in the population of H9/RAR cells the increased number of apoptotic cells could be at least in part connected with increased expression of CD95 (Fas/APO1): in this *RARα *transformed subline CD95 increased almost 10-fold in comparison with parental cell population (from 2,6% in H9 to 21,4% in H9/RAR culture). However in KG-1 and K562 cell populations the number of CD95 expressing cell did not increase after *RARα *transformation.

**Figure 4:**
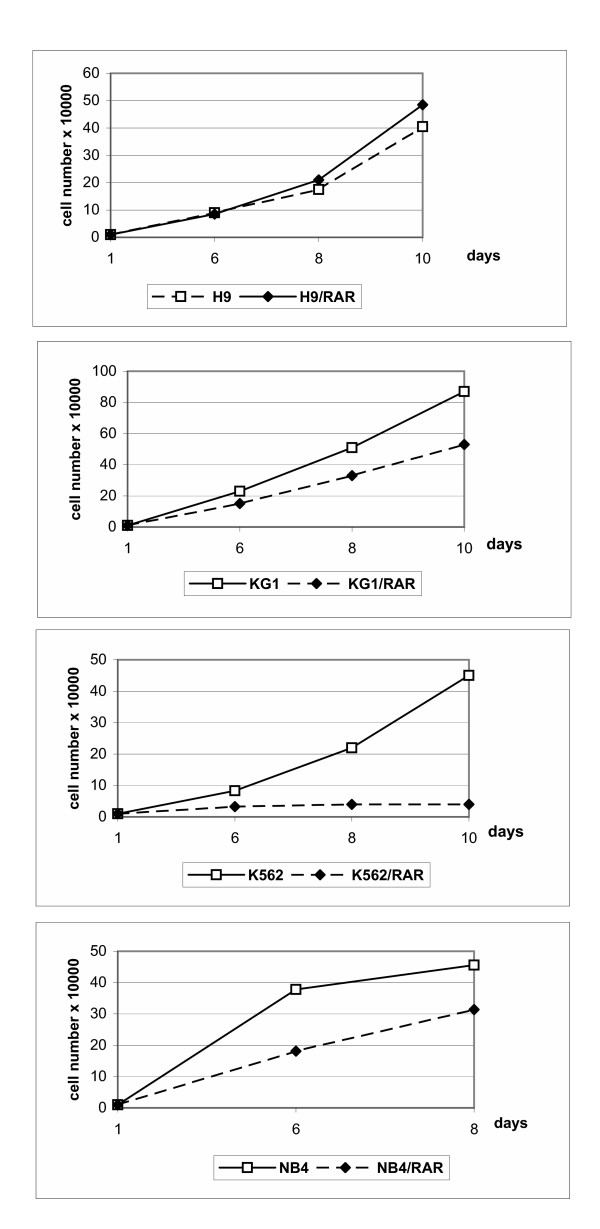
**Proliferation rates of parental and *RARα *infected cells**. Cells were seeded into 24-well plates (1 × 10^4 ^cell per well) and the cell number was counted at days 1,3,5,8 after seeding. This figure is representative of 3 separate experiments.

**Figure 5:**
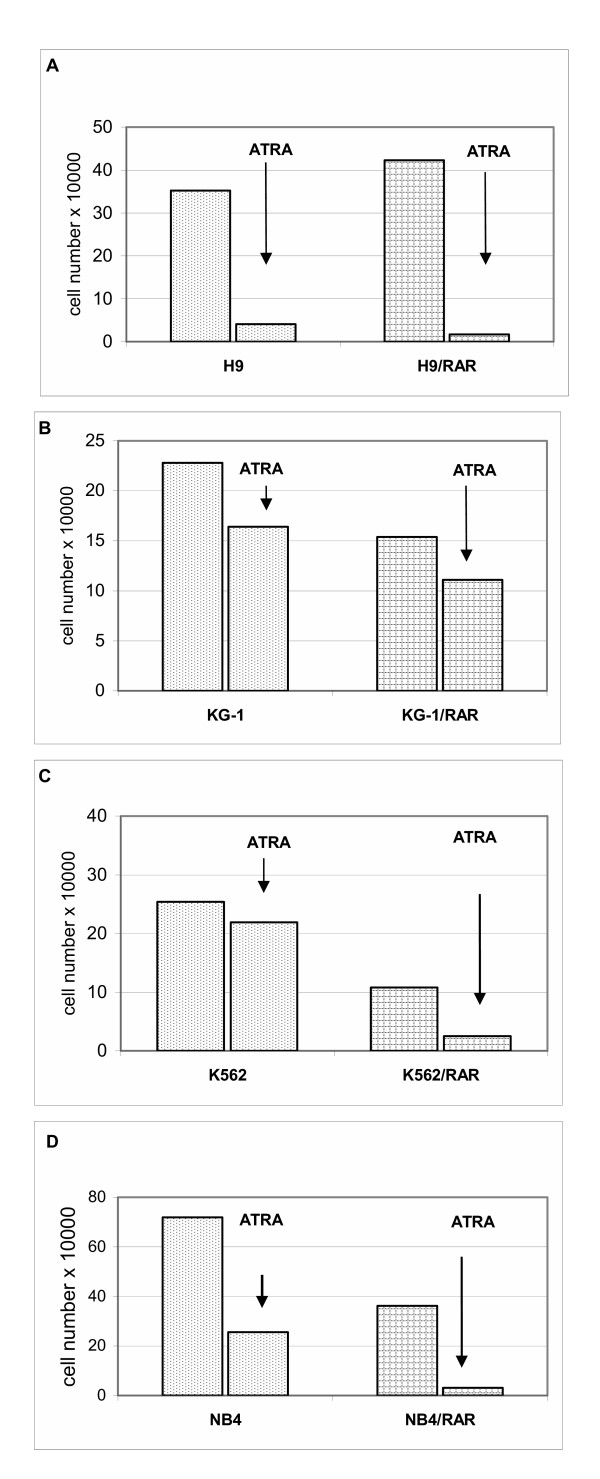
**Influence of retinoic acid (ATRA, 5 μM) on the proliferation of parental and *RARα *transfected cells**. Cells were seeded into 24-well plates (1 × 10^4 ^cell per well), ATRA was added at seeding and the cell number was counted at days 1, 3, 5 and 8 after seeding. This figure is representative of 3 separate experiments.

As Fig. 4 shows, *RARα *transfected KG-1, K562 and NB4 cells proliferated more slowly than parental cells. However H9/RAR cells did not demonstrate slower proliferation rate. Thus, more differentiated status of the *RARα *transformed cell populations was not necessary connected with the decrease in the proliferation rates. All *RARα *transformed cells seem to be more sensitive than wild type cells to inhibitory action of ATRA on cell proliferation (Fig. 5).

### Influence of RARα overexpression on MDR1 gene activity

**Figure 6:**
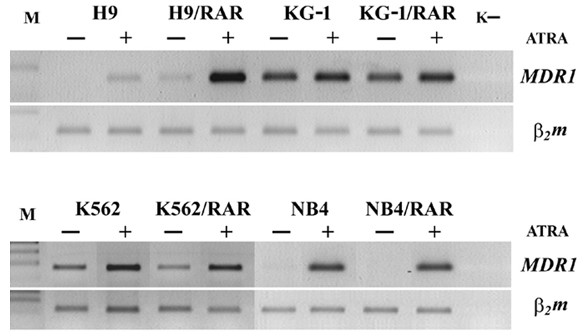
**Intrinsic and retinoic acid induced expression of *MDR1 *gene in parental and *RARα *transfected cells**. k- – water. ATRA (5 μM) was added to cell cultures 24 h after seeding for 48 h. Then the cells were collected and processed as specified in "Methods" (RNA isolation and reverse transcriptase polymerase chain reaction (RT-PCR) analysis of *MDR1 *gene expression). The optimal numbers of PCR cycles were 33 for *MDR1*-specific product for all cells lines except KG1 and KG1/RAR, for these cells the numbers of PCR cycles for *MDR1*-specific product were 26. This figure is representative of 2 separate experiments.

We studied intrinsic and ATRA induced expression of *MDR1 *gene in all cell lines by semi-quantitative RT-PCR technique. The basal levels of *MDR1 *mRNA varied in different wild type cells: in H9 and NB4 cells constitutive *MDR1 *gene expression was not revealed, in K562 wild type cells some *MDR1 *mRNA was found, in KG-1 cells the quantity of *MDR1 *mRNA was large (Fig. 6). It is noteworthy that the optimal number of PCR cycles were 33 for *MDR1*-specific product in all cells while for studies of KG1 and KG1/RAR cells we used 26 PCR cycles. In *RARα *transfected H9 cells the constitutive expression of *MDR1 *gene slightly increased, while in KG-1/RAR and NB4/RAR cells the constitutive *MDR1 *mRNA quantity was not elevated in comparison with the wild type cells, it seems to be even slightly decreased in K562/RAR (Fig. 6). Thus the alterations of the basal level of *MDR1 *expression in *RARα *transformed cells seem to vary in different cell lines.

ATRA (5 μM applied for 48 h) increased *MDR1 *gene expression in all examined cell lines either in parental or *RARα *transfected cells (Fig. 6). In H9/RAR cells effect of ATRA on *MDR1 *expression was significantly greater in comparison with parental cells. In other ATRA treated *RARα *transformed cell sublines *MDR1 *expression was undistinguishable from ATRA treated parental cells (Fig 6).

### Influence of RARα gene transformation on Rh123efflux by the cells

**Figure 7:**
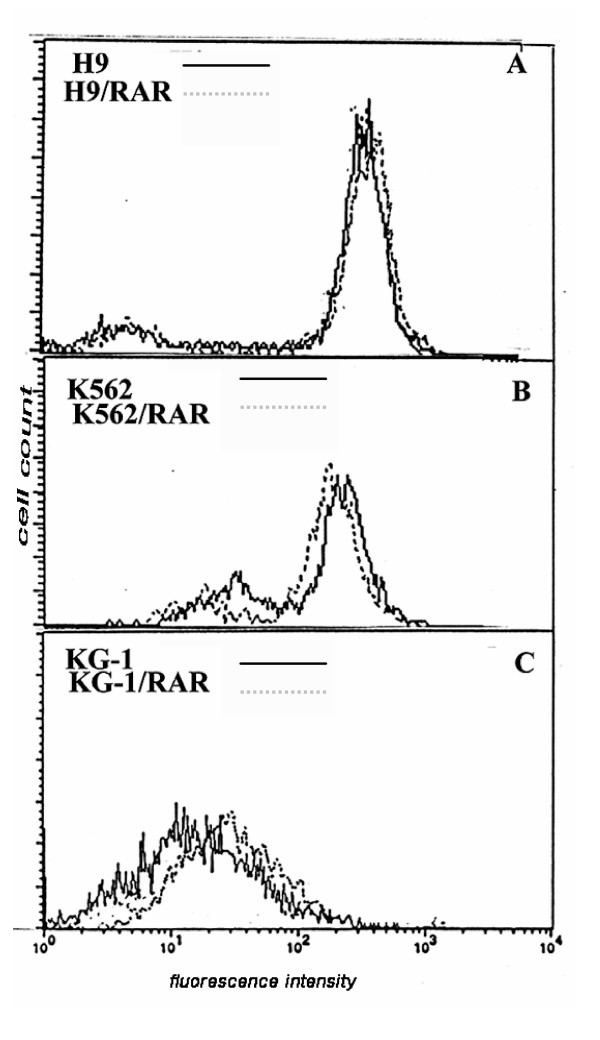
**Evaluation of Rh123 efflux from the parental and cells *RARα *transfected cells**. Cells were loaded with 5 μg/ml Rh123 for 10 min at 37°C, than washed twice with cold PBS, and incubated for 30 min in dye-free medium at 37°C. After the completion of incubation, cell were washed twice with cold PBS. Cell fluorescence was measured on a flow cytometer FACScan (Becton Dikinson, USA). Each measurement counted 5000 events. Non-viable cells were gated out of the analysis on the basis of side scatter. This figure is representative of 2 separate experiments.

The retention of Rh123 by the cells is considered as a test for Pgp functional activity [16,17]. Rh123 efflux from the cells was increased in K562/RAR cells in comparison with the parental cell population (Fig. 7B, Table 2). In H9 and KG-1 *RARα *transformed cells there were alterations in the Rh123 retention (Fig. 7A and 7C): in the populations of H9/RAR and KG-1/RAR cultures the fraction of more dull cells decreased in comparison with parental cultures (mean fluorescence intensity of the cell sublines studied are given in the table 2). This shows that Pgp activity was not elevated in these *RARα *transformed cell populations and suggests that there is some decrease in Pgp functional activity.

**Table 2:** Influence of *RARα *transformation on the intrinsic and induced expression of *MDR1 *gene

	*MDR1 *expression		Rh123 efflux (mean fluorescence intensity)	
	
Cells	intrinsic	ATRA induced	intrinsic	ATRA induced
**H9**	-	+	**450**	**457**
**H9/RAR**	+	++	**506**	**416**
K562	+	++	**446**	**370**
K562/RAR	+	++	**403**	**333**
KG-1	++	+++	**65,7**	**38,3**
KG-1/RAR	++	+++	**90,7**	**20,2**
NB4	-	++	**n.d.**	**n.d.**
NB4/RAR	-	++	**n.d.**	**n.d.**

**Figure 8:**
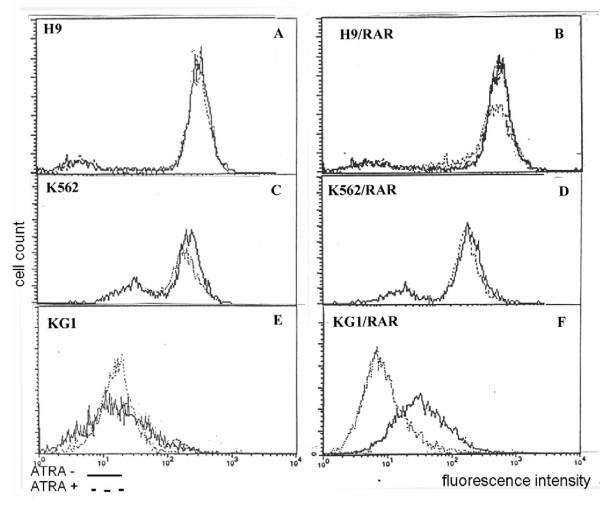
**Influence of retinoic acid (ATRA) on Rh123 efflux from the parental and cells *RARα *transfected cells**. ATRA (5 μM) was added to cell cultures 24 h after seeding for 48 h. Than cells were loaded with 5 μg/ml Rh123 for 10 min at 37°C, than washed twice with cold PBS, and incubated for 30 min in dye-free medium at 37°C. After the completion of incubation, cell were washed twice with cold PBS. Cell fluorescence was measured on a flow cytometer FACScan (Becton Dikinson, USA). Each measurement counted 5000 events. Non-viable cells were gated out of the analysis on the basis of side scatter. This figure is representative of 2 separate experiments.

There was increase in the portion of Rh123 dull cells after ATRA treatment both in K562 and K562/RAR cell populations (mean fluorescence intensity of both populations decreased approximately on 17%) (Fig. 8C, D, Table 2). ATRA induced Rh123 efflux from H9/RAR cells, while in H9 parental population this drug had no effect (Fig. 8A, B, Table 2). In KG-1/RAR cells ATRA induced very prominent increase in the number of Rh123 dull cells (more than 70% decrease of mean fluorescence intensity), while in the parental cell population ATRA decreased mean fluorescence intensity to a lesser extent (Fig. 8E, F, Table 2).

## Discussion

The treatment of leukemia with retinoids is aimed on the induction of leukemic cells differentiation. The question is: are there interconnections between RA/RARα regulated differentiation of leukemic cells and *MDR1*/Pgp activity? In this study we have isolated more differentiated variants of the cultured leukemic cells by the introduction into the cells of *RARα *gene encoding one of RA receptors. All *RARα *transformed leukemic cell populations were characterized by the higher *RARα *gene expression in comparison with the parental cells. All *RARα *transformed leukemic cell populations became more differentiated. This was demonstrated by the studies of the differentiation markers, by the increase in the number of cells dying by spontaneous apoptosis and by the decrease of the proliferation rates of most *RARα *transfected cell sublines. Thus, *RARα *overexpression could result in the increase of the differentiation of various leukemic cell populations.

We compared *MDR1 *gene expression and Pgp functional activity tested by Rh123 retention in parental and *RARα *transformed cells. The results are summarized in the Table 2. Increased constitutive (uninduced) expression of *MDR1 *gene was found in one of four cell lines after *RARα *transformation (H9/RAR, Table 2, Fig. 6). In the previous experiments with melanoma and hepatoblastoma human cells we have shown that constitutive expression of *MDR1 *gene was increased after *RARα *transfection in both *RARα *transformed cell sublines [4]. Thus interconnections between regulation of the basal *MDR1 *and *RARα *activities could exist both in the cells of solid tumors and in the leukemic cells. Our data suggest, that in the cell populations of solid tumors *RARα *overexpression could be accompanied by constitutive *MDR1 *over-expression more often than in the cells of hematopoietic malignancies.

Our study did not reveal the occurrence of the functional Pgp in leukemic cells studied after *RARα *transformation. In H9/RAR cells elevation of the constitutive *MDR1 *expression did not lead to the increase in Rh123 efflux (Fig. 7A, Table 2). Some studies also have described discrepancies between Pgp (protein) or *MDR1 *mRNA expression and Pgp function in leukemic cells [18,19]. These discrepancies could occur for a variety of reasons. Anyway, our data show that increase in the differentiation of leukemic cell populations induced by *RARα *overexpression did not result in the elevation of constitutive Pgp functional activity. In our previous study we found that *RARα *overexpression did not change Pgp functional activity in two *RARα *transformed sublines of human cells (melanoma and hepatoblastoma) but did change it in the rat cells [4]. It seems that exogeneous *RARα *in the cells of human malignancies does not influence basal Pgp functional activity.

In KG-1/RAR characterized by the increased differentiaion (Table 1) we had not found increase in the constitutive *MDR1 *expression and Pgp functional activity decreased (Fig. 7C, Table 2). It is known that blood stem cells and early progenitors expressing CD34 antigen also express high levels of functionally active Pgp [20]. Maturation of these cells is accompanied by the decrease in Pgp expression and even more rapid decrease in Pgp functional activity [21]. It may be suggested that alterations of Pgp function in KG-1/RAR are connected with the differentiation of these cells.

The situation with Pgp functional activity induced by ATRA in the cells studied differs from the situation with constitutive activity of this protein. In all three *RARα *transfected cells ATRA had induced Pgp fuctional activity (Fig. 8. Table 2). Moreover, in two *RARα *transformed sublines (H9/RAR and KG-1/RAR) ATRA activated Pgp, while in the parental cells it had either no effect (H9) or activated Pgp to a lesser extent (KG-1) (Table 2). These data suggest that *RARα *participate in the control of induced, but not in constitutive Pgp functional activity in leukemic cells.

The regulation of *MDR1 *gene transcription and Pgp functional activities are the complex processes [1,2]. The studies of these processes are underway. Our data show that *RARα *gene overexpression could influence the induced Pgp functional activity in leukemic cells, i.e. could participate in the occurrence of multidrug resistance in the populations of these malignant cells. It seems that this influence could depend on the cell context.
